# Supplementary material for: Chronic inflammation was a major predictor and determinant factor of anemia in lactating women in Sidama zone southern Ethiopia: A cross-sectional study
Source: PLoS One. 2020 Oct 5;15(10):e0240254. doi: 10.1371/journal.pone.0240254 (PMC7535025; doi:10.1371/journal.pone.0240254)
Supplement: S1 Data — (PDF) [file pone.0240254.s001.pdf]

Age (years)	BMI (kg/m2)	MUAC (cm)	Household size	School years	Food insecurity	Wealth index	Ferritin (µg/L)	Plasma iron (µmol/L)
24	21.62	27.2	4	3	1	3	16.14	14.8
23	18.99	23.3	6	8	2	5	32.8	27.8
22	23.86	30	4	7	1	1	20.24	11.5
20	22.58	24.3	3	5	1	1	6.67	16.7
20	18.08	22	11	8	1	1	85.14	10.8
20	17.52	21.2	11	8	3	3	0.93	6.4
23	19.23	24	6	3	3	4	0.14	6.3
20	19.87	25	6	3	3	1	255.93	7
32	19.42	24.5	7	0	4	5	11.8	10.5
27	24.25	24.9	6	0	3	4	6.23	16.4
26	21.45	23.8	7	3	4	2	16.28	8.7
20	23.01	28	3	7	1	1	36.07	10.9
22	20.98	25.8	4	4	1	5	8.56	20.1
20	17.95	21.5	8	11	3	4	72.33	20.3
23	18.16	22.6	6	0	1	2	32.17	23.7
30	20.79	26.7	8	0	2	2	12.4	13.2
18	21.56	22.7	3	5	1	1	41.42	25.2
21	19.22	24.1	11	5	2	4	21.44	17.9
24	26.68	28.5	6	7	1	5	70.46	21.4
30	19.56	21	9	0	1	1	16.79	3.1
22	20.91	25.1	4	4	2	5	24.57	11.3
36	26.59	28.5		0	1	5	54.16	17.5
24	21.43	24.4	9	2	4	4	13.57	16.7
27	17.45	21.2		6	1	1	87.98	17.3
20	17.05	20	6	5	3	2	2.17	5.1
20	18.1	22.5	5	5	1	4	103.98	16.9
35	23.15	28.5	9	0	1	1	63.46	11.7
22	20.67	22.5	4	7	1	3	30.04	15.6
20	20.66	23	3	5	1	5	80.13	13
20	21.64	26.3	3	10	3	4	10.94	21.9
22	17.41	23.4	3	1	1	1	11.1	7.4
28	20.57	22.2	3	6	1	5	14.19	10
18	20.45	25.7	14	8	1	4	41.32	15.4
23	24.35	26.3	4	3	1	5	9.14	11.4
21	21.86	26.2	5	3	1	5	25.75	26.2
30	18.9	24.6	7	0	1	1	0.43	9.9
20	20.58	24	11	6	1	3	36.9	17.4
23	20.92	24.6	4	7	1	1	14.5	14.5
25	18.86	24	4	0	3	1	20.26	9.1
28	16.75	21	6	0	4	1	111.43	11.9
26	18.69	21.5	6	9	1	3	29.45	17.8
18	16.52	22	3	6	1	3	3.71	31.9
28	18.56	22.1	7	6	1	5	80.4	18.5
29	19.27	23.2	5	4	3	5	16.79	15.9

20	18.15	22.2	7	0	3	3	6.11	5.4
20	17.29	19.8	4	0	3	1	0	16.1
21	17.06	22.5	5	8	1	4	51.45	18.3
24	23.37	26	3	7	2	4	105.78	10.9
32	18.43	22.3	7	0	3	4	48.01	5
25	20.83	24.5	7	0	3	1	0	12.8
25	21.95	24	6	0	1	2	6.58	8
30	19.71	22.5	8	0	1	1	4.27	5.4
20	22.44	24.2	9	2	4	2	10.61	12.6
23	18.67	21.3	3	1	2	3	7.68	9.5
29	21.14	27	9	0	4	4	39.49	19.3
25	21.97	28	8	0	1	2	37.23	11
28	22.45	25.5	8	5	3	4	51.76	17.4
26	19.33	24.5	8	0	2	2	24.78	26.2
20	24.9	29.5	3	0	1	4	8.23	12.7
20	22.19	25	7	0	3	3	1.16	12
24	23.65	27.8	6	0	2	1	96.33	22.9
20	19.78	23.4	6	2	1	1	122.78	7.9
23	18.56	26	5	10	3	3	15.87	8.9
20	22.8	26.3	6	2	1	3	42.94	14.2
20	19.45	23.7	4	6	1	3	23.86	11.3
20	24.3	26.8	6	4	1	5	12.15	14.7
20	18.49	20.9	5	9	1	2	8.72	22.4
20	18.93	21.3	5	1	3	2	14.95	14.2
20	20.4	24.5	3	5	1	1	19.28	12.2
25	18.71	22.3	8	0	3	2	4.51	9.1
25	20.34	23.3	6	5	4	2	58.8	37.7
25	19.69	24.2	6	0	1	3	0	11.4
28	19.21	21.2	6	3	1	3	61.58	14.3
25	18.66	23.5	7	1	3	4	75.73	18
20	26.2	28.5	5	10	1	5	4.67	11.2
21	21.08	24.5		4	1	3	63.28	25.7
25	21.99	24.7	4	8	1	4	110.45	17.6
25	17.41	21.9	6	0	2	3	6.75	21.5
25	21.66	24.5	9	0	1	2	29.25	11.1
19	18.53	21	7	10	1	4	30.37	10.3
22	20.53	23		0	4	4	17.8	5.4
22	17.53	21	9	8	1	2	23.51	9.7
21	20.68	25.2	7	3	2	5	222.25	19.6
25	24.48	26	6	8	1	3	21.94	11.6
20	20.02	22	4	6	1	4	19.28	21
20	22.13	24.3	4	4	1	3	125.59	16.3
16	18.83	23	3	5	4	3	20.33	32.6
24	21.09	24.7	6	0	1	5	23.51	8.5
19	19.4	22.5	3	6	1	1	3.82	12.3
30	20.64	24.3	7	0	1	2	28.61	7.8
20	21.53	25	4	0	1	1	16.21	8.4

33	23.39	26.3	8	0	1	5	38.35	11.5
20	19.6	24.2	10	11	1	4	3.42	5.7
18	17.87	21.5	3	0	2	3	27.23	10.5
20	20.23	23.4	5	7	1	1	29.72	17.1
22	22.23	25	3	10	1	3	10.66	7.2
19	21.4	25.6	3	4	4	4	25.6	16.2
18	20.34	23.5	3	6	2	2	52.29	27.3
21	20.68	24.5	5	0	4	2	57.05	10.2
23	19.33	23	6	0	3	1	47.72	14.1
19	20.96	24.2	3	10	1	2	8.6	0
25	21.14	25.5	6	5	3	2	32.14	28.1
29	21.56	25.3	9	0	3	4	65.55	20.4
25	23.37	24.1	5	2	3	4	5.94	7
34	21.97	25.1	8	0	3	3	15.17	14.2
36	23.4	27.5	9	0	1	5	28.9	14.7
20	22.13	23.2	3	7	1	2	12.68	14.3
20	19.12	22.4	3	1	1	5	24.77	24.3
26	20.52	22.7	5	2	3	4	31.91	16.8
20	23.1	24.4	3	10	1	2	10.26	22.1
22	20.65	24.3	4	3	3	2	94.7	28.3
20	16.66	22	4	4	1	2	198.86	9.2
24	25.04	26.3	4	6	1	5	89.51	33.3
30	23.43	27	7	0	1	1	95.06	17.9
20	20.72	23.4	4	9	2	5	73.48	28.4
20	16.29	18	4	3	1	2	4.89	22.4
25	17.41	23.3	9	0	3	4	14.87	15.6
18	22.01	24.5	3	6	1	2	75.22	11.9
21	21.74	26.2	3	8	1	5	5.85	12
18	19.29	22.3	3	4	1	3	25.26	16.2
30	16.57	22.5	9	0	1	3	26.8	15.4
18	19.66	23	3	0	1	1	45.77	14
23	19.73	23.6	3	12	1	5	7.38	29.2
22	21.08	25.5	7	0	1	5	32.77	19.3
25	19.07	21.5	6	3	1	4	0	6.2
28	21.03	24.7	5	0	2	3	56.65	7.8
17	24.14	27	3	8	1	5	29.6	6.2
20	22.47	27	5	8	1	4	62.94	21.5
22	19.2	23.9	3	3	1	4	71.07	22.3
21	22.88	26.2	3	8	2	2	27.95	16.8
20	19.93	24	4	2	1	2	48.27	30.7
31	18.18	23.4	8	0	3	5	25.59	10.8
30	25.61	33	9	0	1	1	39.15	17.4
28	22.34	24.2	7	0	4	1	39.7	9.4
23	26.14	30.7	7	0	2	1	221.92	8.3
32	19.12	24.5	7	6	4	3	89.4	18
22	23.75	29.5	3	6	4	5	3.85	5.7
20	25.38	25.5	5	0	4	2	13.19	12.3

20	19.21	24.3	4	7	4	4	54.02	32.9
20	18.89	25.2	4	0	1	4	23.1	10.8
22	27.09	33.5	5	0	4	4	39.38	16
22	20.06	24	7	0	4	1	34.31	16.4
30	19.19	24.2	7	4	3	5	114.81	29.4
22	22.13	23.8	4	10	2	5	23.33	38.4
25	23.06	26.2	7	6	3	3	59.63	18.5
19	21.01	27	5	8	2	5	39.92	30.3
24	18.65	22.8	4	8	2	3	63.15	29.7
27	21.57	26.5	8	0	3	2	12.52	19.7
26	22.7	28.7	8	0	3	3	38.11	21.6
26	20.45	23.1	6	7	3	3	86.62	11.2
18	18.13	23.2	4	10	3	2	64.83	15.3
19	20.99	26	4	6	3	3	36.44	29.1

CRP (mg/L)	AgP (g/L)	Hepcidin (µg/L)	TfR (mg/L)	Selenium (µmol/L)	Zinc (µmol/L)	Hemoglobin (g/L)
1	0.36	7.21	3.8	3	9.1	139
0.4	0.71	5.03	7.1	2.5	8.7	134
0.4	0.32	5.76	8.3	2.8	9.4	131
1.3	0.66	2.31	5.9	3.2	10	141
0	0.97	15.52	4.5	2.6	6.7	139
3	0.7	2.38	8.9	3.6	11.4	115
1.1	0.5	9.17	6.5	3.4	10.7	138
0.5	0.59	19.23	12.7	3.3	9.7	103
0.2	0.57	3.31	7.5	2.4	8.7	123
0.3	0.36	1.78	4.1	2.3	10.3	140
0.5	0.5	11.76	4.5	2.3	9.2	126
0.2	0.58	7.96	6.5	3.7	8.4	133
0.9	0.79	9.66	6.6	4.5	9.8	131
0.5	0.84	11	6.7	3.2	10	141
0.8	0.54	10.67	8.1	3.8	9.4	146
0.1	0.48	7.45	5.5	2.8	8.7	125
0.2	1.24	6.91	4.8	3.1	8	119
0.5	0.56	7.28	4.2	2.4	9	132
0.7	0.54	10.87	5.4	2.6	11.9	140
1.4	1.59	8.22	8.4	3	6.5	105
0.6	0.78	4.62	3.7	3.1	5.7	125
2	0.47	11.46	4.4	2.3	10.4	130
0.2	0.6	13.11	3.6	3.1	9	128
0.3	0.33	16.9	3.8	2.3	8.2	150
1.1	1.9	2.54	14.2	3.5	6.3	94
0.6	1.3	6.7	10.4	3.8	7.8	102
0.7	0.46	9.92	4.4	3.9	10.6	138
0.3	0.68	8.21	6.6	2.6	10.9	138
2.6	0.82	20.47	4.5	2.9	10.3	144
0.5	0.73	10.11	5.7	3.3	11.5	146
0.3	0.74	5.03	12.3	3.1	9.1	111
0.4	0.51	4.55	5.4	3.4	10.9	135
0.1	0.4	8.82	6.2	3.4	8.8	131
0.1	0.7	4.79	6.5	2.4	10.5	136
0.2	0.76	24.27	4.9	2.5	12	140
0.2	0.59	6.03	6.9	2.6	6.9	134
0.4	0.43	4.78	5.8	2.6	10.4	149
0	0.6	5.56	5.5	3	7.9	155
0.1	0.5	8.75	4.2	3	5.8	141
0.2	0.98	12.5	4.5	3.1	9.3	130
0.4	0.56	9.07	3.8	2.4	8.5	122
0.5	0.93	3.34	7.1	3.2	10	147
0.4	1.11	8.21	4.2	2.7	7.6	140
0.1	0.29	5.11	4	4.1	7.6	138

0.2	0.36	3.27	8.8	2.1	10.5	113
6.6	1.7	4.35	8.8	2.2	7.8	102
0.1	0.34	14.34	5.4	3.2	10.4	130
2.1	0.81	14.71	9.1	2.9	12	123
0.4	0.7	2.51	7.8	2.9	8	108
0.5	0.47	8.45	4.9	3.2	9.2	129
0.4	0.43	2.77	8.7	2	9.1	118
5.3	1.06	13.57	5.1	2.6	10.2	144
0.9	0.59	10.18	6	3.6	12.7	136
1.9	1.11	4.24	8.2	3.3	7.4	104
0.2	0.61	15.48	3.7	3.5	9.7	140
0.9	1.08	15.65	4.6	3.5	12.7	147
2.3	0.48	10.77	3.3	2.5	10.3	137
0.4	0.39	10.5	4.5	2.8	10.5	112
2	0.99	4.5	6	3.4	11.7	124
0.8	0.52	4.33	9.9	2.6	7.4	103
0.4	0.41	21.9	5.6	2.7	9.3	114
5.1	1.18	21.67	3.8	2.2	9.1	139
0.5	0.64	3.71	4.6	2.7	9.6	137
0.4	0.72	17.77	3.7	3.7	8.5	128
1.8	0.57	9.29	5.3	1.9	8.4	149
1.2	1.2	8.14	6.3	3.2	12	133
0.6	0.59	9.56	5.3	3.5	10.1	128
0.2	0.61	3.88	3.9	2.8	7.7	109
2.5	0.7	8.59	5.6	3.3	10.4	144
0.4	0.57	3.77	4.2	3.6	12.4	123
0.3	0.57	17.33	3.1	2.4	12.2	139
0.6	0.59	6.23	4.5	2.3	9	123
0.6	0.79	10.4	4.4	3	8.1	118
0.4	0.6	6.69	4.6	2.8	10.4	135
0.1	0.6	2.94	7.4	3.3	10.5	136
0.2	1.02	18.44	2.9	3.1	7.7	141
0	0.37	7.31	3	2.9	7.2	129
0.3	0.47	4.15	4	2.4	7.3	135
1.1	0.61	12.86	5.8	3.6	10.7	127
2.2	0.26	10.79	4.9	3.7	8.4	147
5.7	0.76	18.95	4.8	2.8	9.9	141
0.4	0.41	2.49	6.7	2.3	9.7	120
7.7	0.93	7.18	5.5	3.4	8.6	113
1.3	0.45	6.24	7	4.9	10.2	119
0.4	0.84	11.89	5	3.4	7.1	127
2.9	0.65	6.76	5	3.2	10.2	136
0.7	0.67	1.52	4	1.9	10.4	140
0.2	0.45	2.29	7.1	2.4	9.4	132
0.2	0.44	0.92	9.1	2.5	11.8	135
0.3	0.51	9.34	4	3.2	6.9	129
2.6	0.76	2.09	6.6	3.3	10.9	117

4.9	0.65	5.14	4.9	2.7	8.7	129
0.9	0.71	1.24	9.1	2.3	10.7	124
3	0.83	2.17	6	3.5	9.9	151
0.3	0.53	6.06	3.8	3.2	10.3	134
0	0.7	2.66	6.6	2.7	9.8	125
0.6	0.93	10.44	3.2	3.1	7.4	143
1.3	0.37	11.15	5	3.2	8.8	136
1.2	1.05	7.55	19.4	2.3	9.7	115
3.4	0.76	3.95	4.6	3.4	10.1	129
1.2	2.17	2.85	6.6	0	0	136
1	0.33	5.42	1.4	3.3	8.2	135
4.1	0.98	3.71	4	2.9	8.8	132
0.8	0.46	1.38	7.6	3.7	13	118
0.5	0.9	1.63	7.8	3.7	7.2	128
1.3	0.69	6.22	4.2	4.2	8.8	130
0.7	0.74	0.39	5.3	3.7	7.4	129
0.6	0.86	17.34	4.4	2.9	9.6	134
0.5	1.01	6.84	4.4	3.2	9.7	122
1.2	0.45	0.56	6	2.3	8.3	136
0.5	0.96	10.61	3.5	2.9	8.2	125
5	0.98	25.44	5.1	5	7.8	139
1.2	0.71	8.76	3.3	2.8	8.7	139
0.9	0.41	3.11	5.9	4.1	7.8	133
1.1	0.63	3.71	4.6	3.1	11	128
2.6	1.01	1.66	7.7	3.9	6.3	110
1.4	0.71	10.2	4.3	3.5	11.8	141
5.3	0.84	7.35	8.6	3.3	8.3	119
1.2	0.72	0.87	6.5	2.7	11	133
4.1	0.82	6.31	3.5	3	10.8	141
1.2	1.04	8.48	3.8	3.4	7.8	124
3.1	0.37	8.27	4	3.8	11.8	127
1.8	0.77	1.4	3.5	2.6	11	122
2.7	0.81	1.87	4.3	2.6	8.7	135
24.9	0.94	3.42	4.4	2.1	7.5	111
2.5	0.75	9.28	13.2	2.2	12.4	116
9.2	0.74	16.05	3.1	2.6	9.1	130
2.2	0.44	12.45	6.5	2.8	10	139
1.2	0.81	21.78	4	2.8	9.7	130
0.9	0.66	8.24	6.8	3.4	7.6	139
0.5	0.74	7.88	4.2	4.9	17.5	131
0.4	0.53	2.81	5.2	3.2	8.7	136
1.7	1.31	8.87	5.1	2.9	11.2	147
1.6	0.4	3.63	5.7	3.4	7.2	141
2.3	0.57	17.89	13.2	2.2	8.5	102
1.1	0.75	10.36	4.8	4	7.5	132
40.2	0.99	17.12	8.8	4	9.6	111
3.5	0.99		5.2	3.8	10.7	131

1.6	0.42	6.89	3.1	2.9	11.6	126
1.9	0.75	5.44	4.3	3.3	8.7	132
1.3	0.46	8.41	4.9	3.7	9.6	133
2.3	0.75	6.84	2.9	2.5	9.7	118
0.3	0.36	7.06	2.9	2.7	8.7	132
0.9	0.44	8.87	3.7	3.3	11.2	140
0.7	0.45	8.47	3.5	4.4	10.9	135
2	0.56	7.16	4.9	2.9	9.8	131
6	0.47	4.72	5.1	2.2	10	151
2.9	0.9	5.14	4.5	2.9	10.4	122
1.3	0.63	3.97	11.2	2.6	8.8	120
8	0.68	19.58	3.4	3.7	10.4	128
1.4	1.08	4.56	7.3	2.9	11.3	136
0.8	0.28	6.17	0	3.2	6.6	129
